# The future of health professions education: Emerging trends in the United States

**DOI:** 10.1096/fba.2020-00061

**Published:** 2020-09-23

**Authors:** George E. Thibault

**Affiliations:** ^1^ Daniel D. Federman Professor of Medicine and Medical Education, Emeritus Harvard Medical School Boston Massachusetts USA

## 1 INTRODUCTION

In February 2020 when I was asked to submit some thoughts on trends in the future of health professions education, I had no idea that we were about to experience a once in a century pandemic that would profoundly change health care and the lives and education of health professionals in this country. As I write these personal reflections, we are still in the midst of the COVID‐19 pandemic and cannot yet define what the “new normal” will be for health care, health professional education or society as a whole on the other side of the pandemic. Acknowledging this uncertainty, I believe that the trends I have identified will be more relevant than ever in the post‐COVID world with some specific caveats.

The six trends that I have identified for the future of health professions education are:

1. Interprofessional education in order to better prepare health professionals for future collaborative practice.
2. Longitudinal integrated clinical education that is more patient, community, and chronic disease oriented.
3. Education in the social determinants of health and the social and humanistic missions of the health professions.
4. More emphasis on the continuum of health professions education for the life‐long learning and long‐term well‐being of health professionals.
5. A shift to competency‐based, time variable health professions education to better fulfill our social contract and to produce the most competent practitioners most efficiently.
6. The integration of artificial intelligence and new educational and information technologies into the continuum of health professions education and practice.

It is impossible, of course, to touch on all of the educational issues relevant to every health profession, but I believe I have identified high level trends that will impact all health professions education.

The observations that follow are based on my personal experiences of four decades as a faculty member at Harvard Medical School and several of its affiliated hospitals (Massachusetts General, Brigham and Women's, and West Roxbury Veterans Administration), two decades as a board member and chair of a graduate school of health professions education (the MGH Institute of Health Professions) and a decade leading the only national foundation devoted to improving the nation's health through innovations in health professions education (the Josiah Macy Jr. Foundation).

As I reflect on my five decades in health professions education my observation is that for the first four decades the pace of health‐care delivery reform far exceeded the pace of health professions education reform. The passage of Medicare and Medicaid in the 1960s, advances in science and technology to improve both diagnosis and treatment stimulated by rising NIH budgets in the 1960s and 1970s, increases in the costs of care leading to managed care and mergers and acquisitions in the 1980s and 1990s, the quality improvement and patient safety movements of the 1990s and beyond, and the rise in consumerism with more open access to medical information have all contributed to dramatic changes in the organization and delivery of health care in this country without a parallel transformation in the education of health professionals. Happily, I have witnessed in the past decade a significant openness and willingness to change in health professions education with notable experimentation in both prelicensure (undergraduate) and postlicensure (graduate) education. These changes are heartening, but much more needs to be done to keep pace with this rapidly changing health‐care world and changing societal demographics and expectations.

When I assumed the Presidency of the Macy Foundation in January 2008, I outlined a vision for educational reform that would better align health professional education with societal needs and with an evolving health‐care delivery system. I felt that the health professions education enterprise must not view itself as a closed system in the ivory tower, but as one closely interconnected with the delivery system in which its graduates would work and with the society that they would serve. Health professions education needed to derive its curricular goals from outside rather than inside, and it in turn must be accountable to society in measuring and fulfilling those goals. This framework was very similar to that developed by the Lancet Commission^1^ 2 years later in their work assessing health professions education worldwide. It is a framework that has been adopted by Canada and some western European countries.

The themes to create this alignment became the funding priorities for the Foundation for a decade. The trends that I have identified grew out of this work to align health professions education with societal needs. The trends have been refined and modified based on experience and continuous monitoring of the external environment. In elucidating these trends, I am drawing on very personal experiences as a medical educator and Foundation President.

For each of these six trends I will explain why it is important, provide some examples (drawn heavily from my Macy Foundation experience), identify some challenges, and speculate about the future. I will then conclude with some additional comments about the potential lasting impact of COVID‐19 on health professions education and how the lessons derived from COVID‐19 relate to these trends.

### 1.1 Trend number one: Interprofessional education in order to better prepare health professions for true collaborative practice

The practice of medicine is more and more dependent on teams of professionals caring for complex patients and patients with multiple chronic conditions. Access to reliable, high‐quality primary care is also enhanced by a team approach.^2^, ^3^ There is an increasing body of evidence that care delivered by highly functioning, collaborative teams leads to better patient outcomes. Yet, until recently, health professional education has been designed to keep the professions apart until the completion of the training process. This is in spite of the fact that interprofessional education (IPE) has been written about in the United States since the 1960s, and a 1972 Institute of Medicine Report (“Education for the Health Team”)^4^ strongly recommended IPE.

There are many reasons why IPE did not gain traction in the United States; among these are the logistical obstacles, the strong cultures of each of the professions, the political dominance of physicians who as a group did not embrace IPE, and the lack of a sense of urgency about changing the design of the health‐care delivery system. The tide began to turn in the last decade as several institutions demonstrated that logistical barriers could be overcome, educational leaders in many professions (including MDs) saw the advantages of IPE, and there developed greater urgency about the need for health‐care reform that met the “triple aim” of better health outcomes, better patient experiences, and lower costs. The time was right to assert that the ability to work in a team is a core competency that should be possessed by all health professionals.^5^

I am proud that the Macy Foundation was a leader in this change. In the decade 2008 to 2018 the Macy Foundation supported 44 large grants and 41 small grants with IPE as the primary or secondary theme. In addition, 24 educational innovation projects of Macy Faculty Scholars were interprofessional. IPE was the largest investment that the Macy Foundation made in any of its priority areas. The reasons for the decision to make the investment were that this seemed to be a propitious time to take advantage of the alignment of forces favoring IPE, the belief that IPE could have a large positive impact on improving the health of the public, and the hope that by creating a critical mass of affirmative work we could make this the norm in health professions education. By design, all of the large IPE grants included medical schools and nursing schools. Many included other health professional schools, such as pharmacy, dentistry, public health, and social work. We reasoned that since nurses and doctors were the most numerous and visible of the health professionals caring for patients, changing the culture in those two professions would be models for others.

In reviewing this body of work,^6^ we derived five important lessons that informed our subsequent grant giving. 1) Leadership from the top is essential. Deans, Provosts, Chancellors, and Presidents must embrace IPE and make it a high priority as expressed by budget and organizational structure (such as an office of IPE). Only in this way can the inevitable logistical and political barriers be overcome. 2) Intensive planning with clear educational goals and metrics must lay the groundwork for all IPE initiatives. IPE experiences must be as rigorous as all other parts of the formal curriculum. 3) Interprofessional learners must be engaged through real, meaningful work that advances patient care and their own professional development. These experiences must be reinforced in a developmentally appropriate way throughout the entire educational trajectory. 4) Innovative use of educational technology such as simulation and on‐line, asynchronous learning can help overcome logistical barriers and complement face to face encounters and real patient experiences. 5) Much attention must be paid to faculty development since most faculty have had little or no experience working with faculty or learners from other health professions.

One of the other lessons learned is that IPE is at its best when each profession has the strongest possible educational program—strong uni‐professional education leads to strong inter‐professional education. This is consistent with the experiences of those who have studied successful teams—it is the diversity of points of view and experiences that are brought to bear on the problem that leads to the most successful outcomes. Health science campuses or universities with multiple health science schools that have made IPE a high priority have found that it has helped bring about a cultural change towards greater openness and inclusion that has benefited all faculty and students.

The Health Professions Accreditation Collaborative, which started with Medicine, Nursing, and Pharmacy and now includes 25 entry level health professions education accrediting boards, reports that 22 of its 25 members have or are developing an IPE standard.

So now that IPE is being required by nearly all the health professional prelicensure accrediting bodies (who are also working on a common set of definitions of IPE), can we declare victory and move on? Hardly. There is still great unevenness in the quality, robustness, and penetrance of IPE across all our health professional schools nationally. Free‐standing health professional schools without nursing or medical partners are particularly challenged. We still have more to learn about which are the most meaningful IPE experiences and what are the ideal timing and duration. We also need to solve the challenge of incorporating more IPE in the core clinical experiences of both prelicensure and postlicensure of health professionals.

Almost all of the formal IPE programs to date involve prelicensure health professionals. Though there have been many logistical barriers to overcome to reach our current level of success in prelicensure education, the challenges are even greater in the heterogenous and complex postlicensure world where education takes place virtually entirely in the health‐care delivery system and not in health professional schools. But if IPE is to truly demonstrate a positive impact on the practice of health professionals and the health outcomes of patients, it must become more a part of these later stages of professional development (including what we have called “continuing medical education”). There are some encouraging movements in this direction including a VA primary care program that has medical residents and nurse practitioners sharing practices, a Macy funded pilot study of Ob/Gyn residents and midwifery students training together, and work by the Accreditation Council for Continuing Medical Education (ACCME) to jointly certify interprofessional continuing education programs in nursing, pharmacy and medicine. But much more needs to be done in this arena if our patients are to get the maximum benefit of IPE. This should be an important source of pilot projects and innovations in the future.

Finally, we need to consider the potential contributions of non‐health professionals in a broader definition of IPE. Biomedical Sciences, Engineering, Architecture, Law, Public Policy—to name just a few professions—have important intersections with health and health‐care delivery. There are only a handful of instances that I am aware of in which learners from these professions interact with learners from the health professions, and in each case, it has proven to be beneficial. One can imagine a future real or virtual university where such IPE experiences are more routine.

IPE is here to stay. I regard the last decade as proof of concept. Now that concept needs to be refined, broadened, and linked more closely to improved patient outcomes,

### 1.2 Trend number two: Longitudinal integrated clinical education that is more patient, community and chronic disease oriented.

Since the Flexner Report in 1910 medical school education in the United States has been predominately hospital based and scheduled as a series of rotations on hospital services. As formal graduate medical education (GME) programs for physicians evolved in the decades following the Flexner Report these followed the hospital‐based models. Subsequently, Medicare became the predominant funder of GME in the United States with the payment through the hospital, which reinforced the hospital‐based rotational model. To varying degrees, other health professions have followed this model of hospital‐based rotational clinical education and training.

There are many good reasons why the hospital became the principal site of education in the health professions. The hospital contains the highest concentration of sick patients and this afforded ready access to “teaching material.” It also brought together faculty and learners in one place for increased efficiency of combining teaching and care delivery. As technology and specialization increased, the hospital became even more important as the sole place to have access to all technologies and all specialties. While many of these positive attributes of hospitals continue today, it has been increasingly apparent over the last two decades that there are significant limitations of the hospital and the rotational system as the sole or even principal pedagogical site and method for clinical education in the health professions.

First, the patient population in the hospital of academic medical centers today is less and less representative of the patients that our graduates will care for. Because of both economic and technologic factors hospitals care for only the sickest and most complex patients and for a shorter and shorter period of time. Second, the intensity of care and changes in the schedules of both learners and staff have made it more difficult to accomplish optimal learning environments and achieve educational goals. Third, the rotational model of clinical education lessens the opportunity for learners to appreciate the full impact of illness on patients or to form meaningful relations with patients, faculty, and staff. This is particularly true as logistical and regulatory issues have led to shorter and shorter rotations with more frequent turnover of staff. There is less opportunity for meaningful supervision, assessment, and feedback.

For these reasons, a number of medical schools have piloted and then established a new model for clinical education based on the principles of continuity: continuity of care, continuity of curriculum, and continuity of supervision.^7^

In the full expression of this model, the specialty‐specific rotational clerkships are entirely replaced by a year‐long longitudinal experience that integrates the specialties and emphasizes the care of patients over time with mentoring and supervision by a constant group of faculty. Many of these experiences employ small group problem‐based learning which has been more common in the preclinical than clinical curriculum. A high percentage of the teaching is in the ambulatory setting, but learners also spend time in the hospital when their patients are hospitalized and for certain planned specialty experiences. Some schools have developed hybrid models that retain the traditional specialty clerkships at least in part, and overlay more longitudinal ambulatory and didactic experiences to achieve some of the continuity goals.

The continuity principles are consistent with what we know about successful experiential learning.^8^ The longitudinal integrated clerkship (LIC) permit both horizontal (across disciplines) and vertical (basic science to clinical science) integration and allow for a more planned developmentally appropriate curriculum. Studies comparing LIC students with traditional clerkship students show comparable knowledge and clinical skills in the two groups, but LIC students show greater satisfaction, higher confidence, and a strong sense of patient centeredness.^9^ Perhaps because of markers of more successful professional development, they are more likely to retain the idealism expressed on entry to medical school, which many studies have shown to erode in the clerkship year.

This model has not been as fully tested for other health professions, but one can readily see applicability in other frontline clinical health professions such as nursing, pharmacy, or physician assistant.

In addition to the evidence of improved learner performance and attitudes, there are a number of other potential benefits to a more widespread adoption of this model for at least a portion of health professions clinical education. Many of these relate to the other educational trends discussed in this paper. First, this model creates opportunities for interprofessional learning and the development of team‐based skills, which are much harder to accomplish in short rotations in the intense hospital environment. Second, the appreciation of the impact of an illness on patients over time and the location of the education in ambulatory settings afford more opportunities to understand the social determinants of health and to develop true partnerships with patients and their families. Third, the continuity of the relationship between learner and faculty affords the opportunity to do much more meaningful assessment and give feedback more continuously in the developmental process. This is a prerequisite to achieving competency‐based education. Fourth, the evidence of higher learner satisfaction with the meaningful work they are able to engage in may be at least a partial antidote to the alarming rates of burnout reported among learners in the health professions.^10^ It is also likely that these experiences better prepare them to be life‐long learners. Fifth, there is much concern about the added burden learners place on stressed health‐care delivery sites. Learners in longitudinal experiences can be much more successfully integrated into the workflow of care organizations. Trust can only be developed with time, and with trust comes greater opportunity to make meaningful contributions to the work of the organization in which the learner is embedded.

There are many obstacles to the widespread implementation of the longitudinal integrated clerkship and these include less infrastructure to support teaching in many ambulatory settings, economic pressures for productivity, departmentally based culture and deficiencies in faculty development and incentives for teaching. Pilot programs in a number of institutions have shown that these obstacles can be overcome on a site‐specific basis. In fact, when successful, the LIC model is more popular with both faculty and the host sites. Several new medical schools have been able to institute the LIC model for the entire class, as they have had the advantage of small class size and no prior history of traditional clerkships.

The principles of continuity also should be applied to graduate education, but they will look different than the LIC on the undergraduate or prelicensure level. They may take the form of differentiation into tracks that are tailored to the career goals of the graduate learner. The graduate learner would spend larger blocks of time in specific settings (hospital or ambulatory) that are designed to prepare her for independent practice. This means she would spend less time repeating rotations for which she has already demonstrated competence. In this model the final stages of training look more and more like the beginning of practice, emphasizing the concept of the continuum. The well‐established primary care tracks in many US Internal Medicine programs are an example of this model, but I believe these can be made even more robust and differentiated. In these longer experiences the trainee (about to become practitioner) has the advantage of continuity with patients, site, staff, and mentors.

There will always be a role for shorter, intensive experiences in the hospital or some other technology‐rich site for early learners, graduate learners, and life‐long learners. The ideal educational model will be a blend of experiences designed specifically for the needs of the learners in a developmentally appropriate way. I believe there is growing evidence that some part of the core clinical educational experiences of all prelicensure health professional students should be in a longitudinal experience that is based on the principles of educational continuity.

### 1.3 Trend number three: Education in the social determinants of health and the social and humanistic missions of the health professions

Much of health professions education appropriately focuses on understanding normal human anatomy and physiology, the pathogenesis of disease, diagnostic and therapeutic decision making, and communication skills. It is absolutely essential that every health professional has a keen understanding of the basic and clinical sciences as they pertain to their practice and keep current in them. All of these together contribute to what we call health care. But we realize that health is more than health care. In fact, it has been estimated that all we do in health care contributes about 20% to the health of the public. Larger contributions to health are what have been called the “social determinants of health.” The WHO defines social determinants of health as “the conditions in which people are born, grow, work, live and age, including the health system. These circumstances are shaped by the distribution of money, power and resources….”^11^ Social determinants of health are important not only because they are major contributors to health, but because they also are the principle cause of health disparities (or inequities) that we find in our society. WHO defines health inequities as “the unfair and avoidable differences in health status.” These health inequities have been documented to be prevalent in the US health‐care system.

If the ultimate goal of health professions education is to improve the health of the public (which I believe it is), then one would be incomplete as a health professional without an understanding of the social determinants of health. Therefore, teaching about the social determinants of health should be a part of the education of all health professions.

A recent consensus study of the National Academy of Medicine has provided “A Framework for Education Health Professionals to Address the Social Determinants of Health.”^12^ There are several aspects of this framework which are synchronous with the trends we are discussing in this paper. First, this requires an interprofessional approach to education in order to gain insights from both the direct care health professions (nursing, social work, medicine, etc.) as well as public health and many other professions whose work affects health (architecture, urban planning, law, public policy, clergy to name but a few). Understanding and influencing the social determinants of health requires a collaborative approach. Second, a true understanding of the social determinants of health requires longitudinal and community‐based educational experiences. This reinforces the need for the kind of experiential learning exemplified by the longitudinal integrated clerkships. Third, addressing the social determinants of health requires a commitment to life‐long learning across the whole continuum of the career from the prelicensure learner to fully independent practice.

There are several consequences that will follow from making this commitment to teaching and addressing the social determinants of health. It reminds us that the health professions are at their core humanistic professions, which mean that they place human interests, values, and dignity at the center of their focus.^13^ The health professions are unique among the professions in combining a humanistic heritage and a scientific heritage. In recent decades the scientific heritage has received much more attention, and the challenges of today call for a restoration of the balance. Humanism is elevated not at the expense of science, but to be allied with science so that they together can improve the health of the public.

Addressing the social determinants of health also forces us to confront the issue of diversity and inclusion within our professions and institutions. We cannot dismantle racism, which is one of the most powerful social determinants of health in our society, if we do not exemplify inclusiveness and equity in our own work and organizations.

Addressing the social determinants of health also reminds us that as health professionals we have a social contract. Society has given us special privileges, and in return we are expected to put their interests above our own and use our special knowledge and standing to improve society. We, therefore, have a responsibility not only to understand the social determinants of health, but to help address health inequities. Sometimes we will do this working individually, sometimes through professional organizations, and sometimes through our institutional policies and practices.^14^ Health professionals should learn to be advocates for constructive social change; it is part of our professional responsibility to fulfill our social contract.^15^

Addressing the social determinants of health will better position us to truly partner with patients, families, and communities in linking better interprofessional education and collaborative practice with better health for the public. We cannot achieve better health for the public without these partnerships.^16^

Addressing these goals will not be easy and will require some fundamental changes in our educational processes and the cultures of our institutions. It will require breaking down the silos between the professions and breaking down the walls that have separated the professions from the public we serve. It also will mean introducing new content (social science, humanities, economics) across the continuum of health professions education, and it will require new models for clinical education and community engagement. There have been encouraging movements in these directions in the changes of the last decade. But the pace must accelerate if we are to prepare health professionals who can understand and address the social determinants of health in order to lessen the widening health disparities and improve health for all.

### 1.4 Trend number four: More emphasis on the continuum of health professions education for the life‐long learning and long‐term well‐being of health professionals

Historically each phase of health professional education has been treated separately with different administrative structures, different regulatory structures, and even sometimes different nomenclature. There has been a sense that each phase has the equivalent of a “final exam” and produces a “finished product.” The education of physicians in the United States is an example. There is a clear separation between medical school (undergraduate education) and residency and fellowship (graduate medical education). Undergraduate education occurs within one of 150+ (the number is growing) medical schools in the United States, each with its own administrative structure for education, and nationally it is regulated by the Liaison Committee on Medical Education (LCME) a partnership of the American Medical Association and the Association of American Medical Colleges. The medical school graduate (the “finished product”) then enters into the world of graduate medical education, overseen by over 1,500 hospitals and academic systems as program sponsors, each with their own unique educational administrative structures. Regulation is by the independent Accreditation Council of Graduate Medical Education (ACGME). The “finished products” of this GME system enter practice where they must comply with state licensure laws and hospital/health systems standards. All are required to have some degree of continuing medical education which is administered by a large array of academic institutions, professional associations, delivery systems, and private entities. This enterprise is regulated by the Accreditation Council of Continuing Education (ACCME). Other health professions have similar, if not as complicated, fragmentation of the educational continuum.

The reality is that there is no “final exam,” and there are no “finished” products. Ideally, the health professional is always learning, always in the state of becoming (perhaps that is why we call it “practice”). It is necessary and appropriate that there be milestones and checkpoints along the way to assure the progress of the learner/practitioner and to fulfill our social contract to assure competency. External regulations notwithstanding, it is essential that the attitudes and skills required for life‐long, self‐motivated learning be instilled in all of our learners from the beginning of the educational trajectory. That is what will ultimately assure competency across the continuum.

An important aspect of this is a much greater attention to the quality of the learning environments in which learning and work take place across the life span of the health professional.^17^, ^18^ Without improved environments for learning (and working) other initiatives for educational enhancement and improvement will be for naught. There are many elements to these environments: the personal perspective of the learner, the community in which teaching and learning occur, the organizational culture and practices that surround that learning, and the physical and virtual spaces in which it occurs.^19^ That environment is often shared by learners and practitioners across the whole spectrum of health professions education, which is why the continuum should be the conceptual model.

Another important aspect of the continuum conceptual model and the emphasis on life‐long learning is that it also facilitates the focus on learner and clinician well‐being. There has been an alarming rise in the reported rate of burnout among health professional learners and clinicians, and a recent report of the National Academy of Medicine (“Taking Actions Against Clinician Burnout”) (reference 10) made many concrete recommendations on how system changes can help lessen burnout and promote well‐being. Many of those recommendations deal directly with the learning and working environment. Suboptimal learning environments (across the continuum) contribute to burnout.

Another consequence of this renewed and enhanced emphasis on the continuum, life‐long learning and the learning environment is that this conceptual model is more likely to lead to empowered learners who feel they are doing meaningful work.^20^ Understanding that the ultimate goal of all health professions education is improved health of the patient, the progressive increase in responsibility across the educational continuum enables learners to find purpose in their work and feel like they are making contributions. This is likely another antidote to burnout.

While there are some encouraging movements toward this conceptual model of the continuum of education, this will not be any easy change. Administrative and regulatory structures are well embedded in our system, and there is a lot of territoriality. Academic institutions and health‐care delivery systems need to work more closely together to improve both education and care across the continuum.^21^ And regulatory bodies must work to eliminate the barriers to common language and standards for assessment across the continuum and facilitate smoother and more flexible transitions.

### 1.5 Trend number five: A shift to competency‐based time‐variable health professions education to better fulfill our social contract and produce the most competent practitioners most efficiently

Health professions education has the responsibility to society to produce practitioners who are competent across broad domains of knowledge, attitudes, and skills. Each profession is responsible for establishing its competencies and the educational program to achieve them. All agree that assessment of these competencies is critical in fulfilling our social contract, but historically that assessment has not been rigorous. “Time in place” has often been accepted as a proxy for competency assessment. The required number of months in a given site or discipline and the required number of years in a given program are taken as assurance of competency. This has led to a fragmented and rigid time‐based system of education that does not meet the needs of learners or of the public.

While elements of a competency‐based, time‐variable approach exist within our current educational system, few programs or institutions have fully embraced this model. Two major concepts drive this model: (1) There is a comprehensive curricular, instructional, and assessment strategy based on a framework of observable and assessable competencies derived from patient and societal needs (2) Time is used as an educational resource rather than a limitation or a rule with the consequence that learners and teachers will use time as necessary to achieve the desired competencies.

The connection between the competencies and the needs of society is absolutely central to the success of this model; “competency‐based education begins with an uncompromising focus on translating the needs of contemporary society for improved health care into competencies that must be mastered by health professionals across all disciplines.”^22^ This is an ongoing process across the education/practice continuum and it must be accompanied by robust assessment.

The concept of time as a resource has a liberating effect on both learner and teacher. Learners are allowed adequate time to achieve educational goals but are not required to spend time that is not needed to achieve these goals. Teachers are afforded adequate time for observation, assessment, and coaching to feel comfortable with their judgments. This could result in some learners achieving competencies and moving on in the continuum in less time (and some may take more time). In many instances the total time may be the same, but how that time is used will be different from one learner to another. Thus, the instructional program becomes more individualized, even more so as the learner is farther along the educational trajectory.

This model creates an entirely different dynamic between learners and teachers, and the role of feedback is entirely different than in the traditional model. The learner becomes much more self‐motivated to achieve the competency in order to move to the next level and actively seeks feedback. The teacher becomes the helper and enabler.

There are many challenges in making this paradigm shift, and it will require changes across many domains.^23^ Some of those changes are directly within the control of each educational enterprise, such as curriculum and faculty development reforms. Other changes will involve external bodies for regulatory changes to permit greater flexibility in accrediting programs and certifying individuals. There will need to be more research done to develop more rigorous assessment systems and to evaluate outcomes. Some of the other trends we are discussing in this paper should facilitate this transformation. This work must be interprofessional, emphasize continuity and the continuum of education, and will be facilitated by educational technologies.

Though these changes will be difficult, several programs have demonstrated their feasibility. (reference 23). The Education in Pediatrics Across the Continuum (EPAC) program has successfully integrated undergraduate and graduate medical education for pediatrics in a competency‐based, time‐variable fashion in a pilot program at four US institutions (University of California, San Francisco; University of Colorado; University of Minnesota; and University of Utah). Oregon Health and Science University School of Medicine has implemented a competency‐based, time‐variable curriculum for its entire medical school class. The University of Wisconsin, Milwaukee has a Flex‐Option Program for RN to BSN completion that is competency based and time variable. Queens University in Canada has instituted a competency‐based, time‐variable system for all of its graduate medical education programs. Canada has now made a commitment that all of the GME programs nationally will be competency based and time variable. There are many examples of such programs in Europe and many more pilots underway in the United States.

All of these examples represent “proof of concept” and gives encouragement that both the internal and external changes that are necessary are possible. As in other areas of innovation, the early adopters will pave the way for those that follow.

### 1.6 Trend number six: The integration of artificial intelligence and new educational and information technologies into the continuum of health professions education and practice

Technology is changing in every aspect of our lives, and the pace of that change is accelerating. Health professions education has been slow in adopting new technology, but that pace, too, has now accelerated.^24^ There are now many technologies embedded in our educational system that have improved efficiency and pedagogy and have helped to accomplish other educational goals. Simulation has provided safe and controllable settings for skill development, learning clinical reasoning and developing communication and teamwork skills. It also has been a powerful tool for promoting interprofessional education. Online learning has provided efficient means for knowledge acquisition so that student/faculty time can be more productively spent in higher level functions of interpretation, reasoning, and team skills (the “flipped classroom”). Computerized models have largely replaced cadavers for learning anatomy, and computerized images have largely replaced microscopes in the classroom. Asynchronous, interactive learning has helped to resolve some of the logistic problems with IPE and with distributed models of education and training at multiple sites. Large databases (sometimes obtained from computerized medical records) are being successfully used to direct curricular content and to evaluate educational and clinical performance. All of these changes have helped to improve the education process and also to create closer links between education and our health‐care deliver system, but these changes are small compared with those that are likely to follow.

I will consider three separate aspects of this trend: (1) the increased use of technology as an alternative to traditional education, (2) increased education about technology and artificial intelligence to produce practitioners who are able to successfully use and integrate these tools, and (3) an increased focus on how to capture and utilize time freed by technology to devote to other important functions that cannot be accomplished by technology.

In the realm of technology as an alternate to traditional education, there are now a multitude of online degree and certificate programs in the health professions. This trend will only accelerate as pressures increase to produce more health professionals at a lower cost. There will be an ongoing challenge for quality control and a continued need for faculty development and technological support to adjust to this new educational model. For the clinical disciplines there will always be a need for some real, nonvirtual experiences. More research will be needed to understand the optimal dose and timing of face to face encounters in these “hybrid” models.

Much more time needs to be spent in the future educating and preparing health professionals for a career in which they will be constantly using information technologies and artificial intelligence. By artificial intelligence I mean all of the ways that machines use large data sets to replicate or approximate human cognition. This concept has been around since the 1950s, and for a long time the focus was on the projections that this would someday replace the doctor or other professionals. A more likely scenario is that successful clinicians will harness artificial intelligence to assist them in clinical practice—the two together will be better than either alone. To do that the health professional of tomorrow must have a better understanding of probabilities, confidence intervals, and the use and limitations of large data bases. There is much concern that the algorithms used in AI could actually exacerbate health disparities because of built‐in biases. The health professional of the future must understand the strengths and limitations of these algorithms.

The health professional of tomorrow also will need to be trained in the uses and limitations of telemedicine for both patient and student encounters. All health professional will need training in using the computerized medical record and other technological aids in ways that enhance the patient experience and the patient–clinician relationship rather than detract from them. Our current disappointing experiences with electronic medical records that were developed for business rather than clinical transactions should be a warning to us. Health professionals must be actively involved in developing the systems of the future.

And that brings us to the last aspect of this trend—educators, learners, and clinicians must work together to see that technology enables them at each step along the continuum to devote more time to the higher level functions of reasoning, communication, compassion, and empathy. It will be easy to continue to lament the intrusion of technology or to be nostalgic about the past, but it will take effort and creativity to seize this opportunity to actually elevate the status and role of health professions education and clinical practice. The greatest dividend of the technological revolution will come when all health professionals are freed up to truly “work up to license.” Machine learning can never provide the human touch that all patients want and need in a healing relationship with their clinicians. We must harness technology to enable us to make clinical practice more humanistic.

This sixth trend may in many ways be the most exciting, but it also can be the scariest and most threatening. That is why engagement with the issues should not be delayed, and these concepts must be built into the earliest phases of health professions education and reinforced across the continuum.

### 1.7 Significance of COVID‐19 on these trends

It is, of course, impossible to predict today the long‐term effects of the disruption we are now experiencing from the COVID‐19 pandemic. That disruption has profoundly affected the health of the public with corollary challenges to the health‐care delivery system and health professions education. Beyond that, the economic, social, and psychological effects on society are likely to be felt for years, if not decades. But with those caveats, I will posit that it is likely that the COVID‐19 experience will actually reinforce and accelerate these trends I have identified. I will also note some other issues it has raised about the preparation of health professionals for the future.

First, as to COVID‐19 and the trends. It is quite clear that the enormous stress placed on our health‐care delivery and public health systems could not have been dealt with without a collaborative and interprofessional approach. The daily heroic stories of frontline health workers have stressed the interdependence of the team. If we ever had any doubt that we are preparing health‐care workers of the future to work in teams, the COVID‐19 story has put that doubt to rest.

Regarding the second trend, the disruption in hospital‐based education during the pandemic has been profound. For all practical purposes clinical education in the hospital stopped. This was done to protect the learners and to conserve scarce personal protective equipment, but also because of the realization that the COVID‐19 hospital was not an environment conducive to education. On the other hand, ambulatory education did continue in many settings, and students who have acquired both the trust and skills that are part of a successful longitudinal integrated experience were actually able to be helpful to their patients and the care sites in these stressful times. I received a personal communication from one of the leaders of these experiences with the following observation.^25^ “LICs are proving particularly resilient and beneficial in the time of Covid. Indeed, I keep learning of stories in the United States, and in other countries, of how LIC students are able to continue to benefit their patients, preceptors, offices/institutions, and communities precisely because of the model—with the LIC model, students are known and trusted and the students know their patients and clinical microsystem so well. All this is to say that on top of all the proven educational benefits over these many years, we now see that the power of time affords the trust, connectivity, systems training, patient–preceptor–system relationships need to address current COVID needs and the likely care delivery that is coming. LICs create time and relationships AND flexibility and these offer enormous benefits for education and service.”

The pandemic has also highlighted the importance of the social determinants of health because of the striking differences in outcome based on race, ethnicity, economic status, and zip code. The relationship between social factors (racism, housing, job, transportation, air quality, access to care) and health outcomes has never been clearer or starker. COVID‐19 has called for us to not only better understand these relationships but to do something about them. And health‐care professionals must be central to that discussion and action.

The importance of the environments in which we work and learn and the importance of focusing on the long‐term well‐being and resilience of our health professionals have also been drawn in sharp relief by the pandemic. The other side of the coin of the heroism of health‐care workers has been the effect of this continually stressful environment on increasing the likelihood of burnout, depression, and suicide. We will not know for some time the long‐term psychological and morale consequences of the pandemic on health‐care workers, but we will need to pay more even more attention to these environmental factors at each point in the education and clinical continuum going forward. The extraordinary humanism shown by our health professionals must be returned in kind by developing and maintaining humanistic systems of care and learning.

The stress on the whole health‐care system showed the importance of assuring competence at all levels of the health professions and also of assuring that we have enough health professionals with the right skills in the right places. That is, after all, our social contract.

Finally, the pandemic has shown very clearly the increasing role that technology will play in education and care. Most health professional schools went to entirely online learning, and that is likely to continue in some fashion into the next academic year. In the clinics, a high percentage of visits become telemedicine visits. This enforced rapid transition in both these domains is likely to lead to rapid improvement in and acceptance of these technologies, and I expect some of these changes will be permanent.

So, each of the trends has been reinforced and I suspect accelerated by the pandemic. As traumatic as has been this disruption, we may look back at it as an accelerant of change albeit at a very high price.

There are some other changes that also are likely to stay that were not part of the trends I have identified. There must be a greater emphasis going forward in health professional education on emergency preparedness, with the likelihood that other epidemics and pandemics will occur in the professional lives of all of our trainees. Also, the pandemic has reminded us of the enormous importance of public health and epidemiology in our health‐care system. This has profound implications at a policy level because we have so woefully underinvested in public health and public health planning. But it also has implications at the education and practice level in that we must much more actively integrate these disciplines with the other health professions—consistent with the interprofessional education trend.

## 2 CONCLUSION

This is an exciting time in health professions education. Building on a decade of innovations that provided proofs of concept and some guiding principles, we are poised for a decade of explosive innovation along the six trends outlined. It is good that we are ready for this, because the public we serve desperately needs these changes to enable it to achieve the health that is our goal.

It should also be apparent that these trends are not totally independent from one another; they are, indeed, interconnected. Interprofessional education helps to create the culture for addressing the social determinants of health and life‐long sustaining learning environments. Longitudinal integrated clinical experiences facilitate insights into the social determinants of health and create the continuity environment for competency‐based assessment and professional development. Educational technologies and big data, properly harnessed, can help promote all of these changes. These are but a few examples of the interconnectedness of the trends, and illustrate why these changes need to be done together.

All of these changes together will in fact be needed if we want to produce the health professionals we need for an optimal health‐care system and a healthy public. This will require leadership and culture change. We must break down the barriers that separate the professions and the barriers that separate education from health‐care delivery and that separate both from the patients, families, and communities we serve. We must remember that health professions education and health‐care delivery both have the same goal—the improved health of the public. That will only happen if we produce health‐care professionals who are truly collaborative, community oriented, cognizant of the social determinants of health, resilient, competent life‐long learners who are adept at harnessing technology to serve their patients, and who possess empathy and compassion. In other words, they model the ideal blend of humanism and science. It is a tall order, but we can do it.
